# Genome-Wide Association Study and Genomic Predictions for Hydroxycinnamate Concentrations in Maize Stover

**DOI:** 10.1021/acs.jafc.4c07467

**Published:** 2025-01-13

**Authors:** Noemi Gesteiro, Rosa A. Malvar, Ana Butrón, James B. Holland, Xosé C. Souto, Ana López-Malvar, Rogelio Santiago

**Affiliations:** † UA MBG-UVIGO, Misión Biológica de Galicia (CSIC), Pazo de Salcedo, Pontevedra 36143, España; ‡ U.S. Department of Agriculture-Agricultural Research Service, Plant Science Research Unit, Raleigh, North Carolina 27695, United States; § E.E. Forestal, Dpto. Ingeniería Recursos Naturales y Medio Ambiente, Pontevedra 36005, Spain; ∥ Facultad de Biología, Dept. Biología Vegetal & Ciencias Suelo, Unidad Asociada MBG-UVIGO, Universidad de Vigo, Lagoas Marcosende, Vigo 36310, España

**Keywords:** *p*-coumarate, ferulate, resistance, digestibility, biofuel, genomic prediction

## Abstract

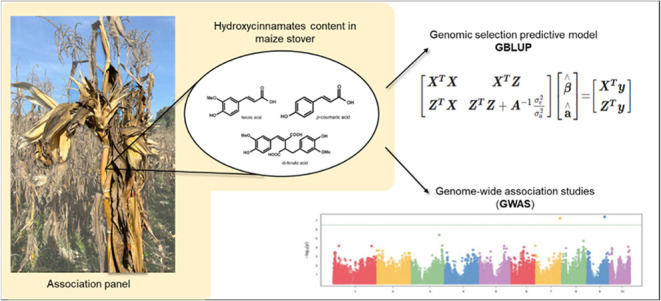

Hydroxycinnamates, like ferulate (FA) and *p*-coumarate (*p*CA), are important components of maize cell walls, which influence pest resistance, ruminal digestibility, and biofuel production. Increasing their concentration has been linked to increased pest resistance, but also may lead to a decrease in nutritional value or bioethanol production efficiency. Therefore, improving forage quality or biofuel production without compromising plant resistance and a thorough understanding of the biosynthesis and deposition of these compounds is necessary, especially in stover, which is the feedstock for second-generation biofuel production and determines animal forage quality. This study aimed to identify genomic regions associated with hydroxycinnamates and to develop genomic prediction models to determine the best selection approach to modify hydroxycinnamate content. Although heritability estimates for hydroxycinnamates were moderate, direct phenotypic selection is discouraged because hydroxycinnamate quantification is laborious and time-consuming. Negative genotypic correlations were observed between animal digestibility and *p*CA content and positive with diferulates content, suggesting differing effects compared to previous studies on maize pith. However, no colocalizations with digestibility QTLs were found, highlighting the need for further research. Given the moderate predictive capacity of GBLUP prediction models, genotypic selection is proposed as the most promising alternative for modifying hydroxycinnamate content.

## Introduction

Hydroxycinnamates, such as ferulate (FA) and *p*-coumarate (*p*CA), are essential cell wall components in a variety of grasses including maize. Originating from the phenylpropanoid pathway, derived from phenylalanine and tyrosine, they play crucial roles in cell wall structure and functionality.^1^ Ferulic acid is mainly esterified on the arabinosyl residues of the arabinoxyl chains, although it can also be linked by ester-linked to the lignin polymer^2^ and can be coupled by oxidative bonding to form a variety of dimers (DFA), cross-linking polysaccharide chains. Total diferulates (DFAT) usually refer to the sum of 8-*O*-4, 5–5, and 8–5 diferulates, but other diferulates are also present in the maize cell wall in smaller proportions.^3,4^ On the other hand, *p*CA is esterified at the γ position of the side chains of the S-units of lignin, contributing to their deposition and affecting cell wall strength. In addition, small amounts of *p*CA can be bound to arabinoxylan.^2,5^

Hydroxycinnamate content has been widely associated with economically important traits, such as pest resistance, ruminant digestibility, and biofuel production.^6−9^ Higher levels of cell wall-bound hydroxycinnamates in maize kernels, leaves, or stalk tissues have been associated with increased levels of pest and disease resistance,^8−11^ but have been also associated with decreased maize cell wall digestibility by increasing cell wall recalcitrance through cross-linking.^12,13^ In addition, colocalization of QTLs for pest resistance, animal digestibility, and bioethanol production with hydroxycinnamate content have been reported.^6,8,14−16^ Therefore, reduction of cell wall FA and DFA cross-linkages have become a key target in the improvement of maize as a forage crop to increase digestibility in ruminant livestock.^13,17^ Similarly, reduction of the ferulate cross-links that form that protective matrix around the cellulosic skeleton of the cell wall has been suggested as a promising strategy to improve the degradability of maize lignocellulosic biomass during the ethanol production process.^18,19^

Therefore, the content of hydroxycinnamates bound to the cell wall could be proposed as an indirect selection trait for improving the nutritional quality of maize forage. However, the accumulation and participation of these compounds in maize cell wall properties is complex and differs greatly from one genotype to another, from one plant tissue to another, and even within the same tissue, from one stage of development to another.^8^ Stover or lignocellulosic feedstock, the residues left after harvesting the grain, is a key tissue, as it is the largest and most readily available substrate for producing second-generation biofuels, defining the quality of animal forage.^6,18^ Therefore, improving biofuel or silage production more efficiently and cost-effectively, without compromising plant growth and resistance, requires studying the genetics involved in the biosynthesis and deposition of cell wall hydroxycinnamates particularly in the stover (stem and leaves).

In this context, it is important to study the heritability and genomic regions involved in inheritance before addressing the role of these traits in maize breeding. Improving hydroxycinnamate concentration by phenotypic selection is a slow and laborious process due to the expense, time, and labor required for sample processing and chemical analyses. Another option for including hydroxycinnamate as a selection target could be marked-assisted selection or genomic selection. However, for marker-assisted selection to be more efficient than phenotypic selection, it is crucial that both the precision of the identified QTLs and the proportion of explained genotypic variance are high. Previous studies have revealed a large number of QTL involved in cell wall-bound hydroxycinnamates, each one explaining a low percentage of phenotypic variability.^15^ Given those limitations, it is suggested that genomic selection could be a promising option to phenotypic or marker-assisted selection. Genomic selection, based on the use of a large number of DNA markers to predict breeding values, has revolutionized plant breeding by allowing an accurate and early assessment of the genetic potential of inbreds.^20,21^ Previous studies have shown that this selection strategy can be promising even when the accuracy of prediction models is moderate.^22^

The use of diverse inbred panels for genome-wide association studies (GWAS) is a step forward since it increases diversity beyond biparental and multiparental populations, facilitating the mapping of QTLs in much more specific genomic regions, and also allowing the possibility of identifying relevant candidate genes. In this context, López-Malvar et al.^16^ evaluated a diversity panel of 270 inbreds to study hydroxycinnamates bound to the esterified cell wall in pith internodes, identifying 22 QTLs. The panel used by López-Malvar has proven successful in characterizing several traits through a candidate gene strategy in previous studies.^23−25^ The present study is a subset of the Ames association panel (North Central Plant Introduction Station), which also has proven useful for candidate gene identification through GWAS.^26^ The Ames panel comprises 2711 inbred lines and has a higher diversity than that used in previous research on hydroxycinnamates. It includes varieties from breeding programs with diverse objectives and environments, ensuring a broadly diverse gene pool. A subset of 835 inbred lines has been selected for phenotypic evaluation, although only 350 performed well under our environmental conditions. Furthermore, unlike López-Malvar et al.,^16^ we have targeted stover rather than specific internodes, as this material is crucial for several uses that need to be prioritized in a more sustainable agriculture.

The objectives of this study are to identify genomic regions involved in the cell wall-bound hydroxycinnamate concentration, particularly in maize stover, and to move forward in the development of genomic prediction models for these compounds. The ultimate goal is to determine the best breeding method for modifying hydroxycinnamate concentration in order to indirectly improve the quality of stover for bioethanol or forage production without compromising plant resistance to biotic stresses.

## Material and Methods

### Plant Material and Experimental Design

A subset of 350 inbreds from the USDA-ARS NCRPIS (North Central Regional Plant Introduction Station, Ames, Iowa) association panel was utilized in the current study. A larger set of 835 lines of the Ames panel was evaluated in Pontevedra (42.240° N, 8.380° W, at an altitude of 20 m above sea level) in 2018 and 2019 using an augmented design with 17 blocks in 2018 and 14 in 2019;^27^ each block included 50 nonrepeated inbreds from the panel and six representative inbred checks used in the temperate zone of Europe (A619, A632, A662, A665, PH207, EP42). Each experimental plot consisted of a single row 2.4 m long with 13 plants planted. The distance between consecutive plants in a row was 0.21 m, and a spacing of 0.8 m between rows was maintained, resulting in a planting density of approximately 60,000 plants/ha.

The 350 inbred lines were selected from the larger set for hydroxycinnamate concentration evaluation based on a good plant stand, adequate development, and wide variation for flowering. These inbred lines were divided into five maturity groups based on the timing of female flowering, defined as the time when 50% of the plants exhibited visible silks. Stover samples were collected on five different dates, as each genotype was harvested approximately 70 days after flowering. The entire above-ground portion of the plant was collected from 350 selected inbreds and six inbred controls. However, from only 238 inbred did we have sufficient material in both years to conduct biochemical analyses. The samples were dried at 60 °C for 7 days, ground using a Restch SM100 cutting mill, and subsequently reprocessed in a Fritsch-Pulverisette 14 mill with a 0.75 mm mesh sieve.

### Laboratory Analysis

For the quantification of esterified hydroxycinnamates, an updated method by Santiago et al.^28^ was followed. The extraction was performed with 30 mL of MeOH (80%) per 1 g of sample and shaken in the dark for 1 h. Then, it was centrifuged and 20 mL of NaOH (2M) was added to the pellet under nitrogen atmosphere and shaken for 4 h. The pH was adjusted to 2 with HCl (6M) and/or NaOH (6M), and the mixture was centrifuged again. The pellet was washed with distilled water, and two liquid–liquid extractions were performed with ethyl acetate (50 and 30 mL). The mixture was dried in a rotary evaporator for 8 h, resuspended in 1.5 mL of high purity MeOH, and filtered for final HPLC analysis.

Analyses were performed using a Waters HPLC 2690 system (Waters, Milford, MA) with a separation module, a Waters 996 diode array detector, and a YMC ODS-AM column (Waters) (100 mm × 2 mm internal diameter; 3 μm particle size). Elution conditions included a mobile phase of acetonitrile (solvent A) and 0.05% trifluoroacetic acid in water (solvent B) with the following program: initial conditions 10:90 (solvent A/B), linearly changing to 30:70 (solvent A/B) in 3.5 min, 32:68 (solvent A/B) in 6.5 min, 100:0 (solvent A/B) in 4 min, an isocratic solution of 100:0 (solvent A/B) for 4.5 min, and finally returning to initial conditions in 3 min. The flow rate of the mobile phase was 0.3 mL·min^–1^, the total analysis time was 21.5 min, and the sample injection volume was 4 μL. Quantification was performed at 325 nm. The content of coumarate (*p*CA) and ferulate (FA) monomers and the content of ferulic acid dimers were quantified: DFA 5–5 (4–4′-dehydroxyl-5–5′-dimethoxy-3′-bicinnamic acid), DFA 8-*O*-4 ((Z)-β-4-hydroxy-3-methoxycinnamic acid), and DFA 8–5 which was calculated as the sum of the 8–5-open or linear forms (4–4′-dihydroxyl-3, 5′-dimethoxy-β,3′-bicinnamic acid) and 8–5-benzofuran or cyclic (trans-5-((E)-2-carboxyvinyl)-2-(4-hydroxy-3-methoxyphenyl)-7-methoxy-2,3-dihydrobenzofuran-3-carboxylic acid). The total content of diferulates (DFAT) was calculated as the sum of the following three DFA isomers: DFA 8-*O*-4, DFA 5–5, and DFA 8–5. The standard recovery with this procedure was 96–98%.

Retention times were compared with freshly prepared standard solutions of p-coumaric and ferulic acids (Sigma-Aldrich Quimica SL, Madrid, Spain) and of DFA 5–5 synthesized by Dr. John Ralph’s group (Department of Biochemistry, University of Wisconsin, Madison, WI), and were as follows: pCA = 5.34 min, FA = 6.05 min, DFA 8–5 open = 6.60 min, DFA 5–5 = 7.66 min, DFA 8-*O*-4 = 8.51 min, and DFA 8–5 benzofuran = 8.72 min. The identities of FA dimers were confirmed by a comparison with the authentic 5–5 standard or previously published retention times and UV spectra.^29,30^ Calibration curves for the standards were built and used for external quantitation as follows: PCA: *Y* = 5.42e + 004*x* + 2.26e + 004 (*R*^2^ = 0.9971), FA: *Y* = 3.92e + 004*x* + 1.01e + 005 (*R*^2^ = 0.9981), DFAs: *Y* = 4.98e + 004*x* + 3.42e + 004 (*R*^2^ = 0.9933). The baseline concentration for detection was 2 μ/mL.

### Genotyping

The Ames panel lines were genotyped^26^ using the genotyping-by-sequencing (GBS) method at the Institute of Biotechnology, Cornell University. Data for approximately 1,000,000 SNPs are publicly available on Panzea (ZeaGBSv27_AGPv4) (http://www.panzea.org, accessed on July 28, 2023). To construct the genotypic matrix for the inheritance and GWAS study of the entire panel, initially, a subset of 350 lines was extracted. Subsequently, this subset was further filtered, selecting 238 lines with phenotypic data for both years of the study. Additionally, a filter was applied to the genotypic data of these 238 lines, excluding markers with more than 20% missing data (with heterozygous genotypes considered as missing data), those with a minor allele frequency (MAF) less than 0.05, and monomorphic and multiallelic SNPs and insertion/deletion polymorphisms (INDELs). This filtering was performed using TASSEL 5.2.54 software,^31^ resulting in a final genotypic data matrix comprising 238 lines for 153,009 SNPs. For genotypic selection, a stricter filter based on the percentage of missing data was applied, reducing it to 5%, resulting in a final set of 25,422 SNPs. To address the missing data, the *A.mat* function from the rrBLUP package in R was used.^32^ This function replaces missing marker values with the population mean for the respective marker.

### Statistical Analysis

A combined analysis of variance was performed for the traits studied using the PROC MIXED procedure of the SAS software.^33^ Best Linear Unbiased Estimators (BLUEs) were calculated for each inbred across years. Genotypes were considered as fixed effect, while years, nested block within year, and the interaction between year and genotype were considered as random. Mean comparisons were carried out using the Fisher’s protected Least Significant Difference (LSD) method. Trait heritabilities were estimated on a plot basis following the method described by Holland et al.^34^

### Genotypic Correlation

Genetic correlation coefficients were calculated between cell wall hydroxycinnamates and grain yield, digestibility of the organic matter *in vitro* (DMO), and saccharification efficiency (SAC) using REML estimates according to a mixed model procedure published by SAS.^35^ The yield, DMO, and SAC data utilized were obtained from previous studies with the same samples.^36,37^ Yield was calculated as grain weight per plant and adjusted to 14% grain moisture. DMO was determined by near-infrared reflectance (NIR) spectroscopy. Spectral data were collected from dried and ground samples (<1 mm) using a Foss NIRSystem 6500 spectrophotometer (Foss NIRSystem, Silver Spring, Washington), and analyzed with WinISI II v. 1.5 software (Infrasoft International, Port Matilda, PA, USA). Outlier samples were identified using the Mahalanobis global distance^38^ and analyzed in the laboratory to improve NIR predictions. DMO was determined following the procedure described by Tilley and Terry,^39^ modified by Alexander and McGowan.^40^ SAC was determined as described by Gomez et al.^41^ Samples were pretreated with 0.5 M NaOH at 90 °C for 30 min, washed four times with 500 μL of sodium acetate buffer, and finally subjected to enzymatic digestion (Celluclast 2, 7 FPU/g) at 50 °C for 9 h. The amount of sugars released was evaluated against a standard glucose curve using the 3-methyl-2-benzothiazolinone hydrazone method.^42^

### Association Analysis

The GWAS analysis was conducted using the BLUEs. GWAS analysis was performed with Bayesian-information and linkage-disequilibrium Iteratively Nested Keyway (BLINK) method, which is based on a linear mixed model and implemented in R.^43^ Unlike some traditional approaches, BLINK eliminates the need for markers to be evenly distributed across the genome, replacing this assumption with linkage disequilibrium information.^43^ The association between a trait and a SNP was considered significant when the *p*-value was lower than the Bonferroni threshold assuming an experimental error of 0.05/*m*, where *m* is the number of markers.

To determine the confidence intervals for each significant SNP, a linkage disequilibrium (LD) study was conducted in windows of ±10 Mbp around each significant SNP. First, LD was calculated by squaring the correlation coefficient (*r*^2^) between all marker pairs within the window using TASSEL 5.2.24 software.^31^ Subsequently, the r^2^ data were plotted against the distance in base pairs. Then, the drift-recombination model was utilized to fit a nonlinear regression of the r^2^ expectation. To perform this fitting, an R script developed by Marroni et al.,^44^ based on an equation previously described by Remington et al.,^45^ was used. The bounds of the confidence intervals around the significant QTLs were defined according to the ± distance at which LD decayed bellow *r*^2^ < 0.2.

The search for candidate genes was performed within these SNP confidence intervals. Genes contained within the confidence intervals were identified and characterized according to the B73 maize reference genome set available in the MaizeGDB browser.^46^ The search for candidate genes was performed on the B73 version 5 sequence (Zm-B73-REFERENCE-NAM-5.0).

### Genomic Prediction

Genomic prediction was conducted using the GBLUP (Genomic Best Linear Unbiased Prediction) model.^47^ To perform the calculations, the *mixed.solve* function from the rrBLUP package in the R programming environment was employed.^32^ This involved calculating the genomic relationship matrix (G) among the evaluated lines based on the genetic markers and utilizing this matrix to predict the breeding values (GEBV), estimating genetic effects for each line, thereby allowing us to predict the phenotypic value (BLUE) for the concentration of hydroxycinnamates in each line.

Cross-validation with 10 iterations was used to assess the accuracy of genomic predictions.^48^ For this process, the data were divided into 10 groups of approximately equal size. Eight of these groups consisted of 24 individuals each, while the remaining two groups contained 23 individuals each. Each group was used once as the test set in the analysis, while the other nine groups served as the training set. This cross-validation process was repeated 10 times with a different test set selected in each iteration and the rest of the data used as the training set. At the end of the 10 iterations, the average results and error obtained for each test set were calculated. The estimated correlation between the observed and predicted values for the testing set provided an unbiased measure of the model’s prediction ability. Prediction accuracy is the prediction ability divided by the square root of heritability.^49^

## Results

### Analysis of Variance and Heritabilities

Significant differences among the panel inbreds were observed (*p* < 0.0001) for all quantified cell wall-bound hydroxycinnamates. Significant genotype × environment interactions were observed for pCA (*Z* = 2.73, *p* = 0.003), but not for FA (*Z* = 1.32, *p* = 0.093) or DFAs (*Z* = 0.95, *p* = 0.171 for DFA 5–5; *Z* = 1.28, *p* = 0.101 for DFA 8-*O*-4; *Z* = 0.41, *p* = 0.339 for DFA 8–5; and *Z* = 0.39, *p* = 0.347 for DFAT). Regarding estimated heritability, it was observed that pCA and FA exhibited reasonably high heritability, while individual and total diferulates showed moderate heritability (Table 1).

**Table 1:** Mean, Range, Least Significant Difference (LSD), and Heritability (*h*^2^) for the Cell Wall-Bound Hydroxycinnamates in Maize Stover of 238 Inbreds from the Ames Association Panel Evaluated in Pontevedra in 2018 and 2019

trait	mean	range	LSD^a^	*h*^2^ ± SD^b^
*p*-coumarate (mg/g)	7.55	4.01–11.13	0.89	0.67 ± 0.04
ferulate (mg/g)	3.17	1.82–4.55	0.45	0.64 ± 0.05
DFA 5–5 (mg/g)	0.18	0.07–0.29	0.04	0.52 ± 0.06
DFA 8-*O*-4 (mg/g)	0.23	0.08–0.51	0.04	0.45 ± 0.07
DFA 8–5 (mg/g)	0.26	0.13–0.50	0.07	0.48 ± 0.06
total diferulates (mg/g)	0.68	0.32–1.3	0.11	0.47 ± 0.07

a Least significant difference (*p* < 0.05).

b Heritability is considered significantly different from zero when it is 2 times greater than the standard deviation (SD).

### Genotypic Correlation

High positive genotypic correlations were estimated among FA, DFAT, and individual dimers. However, only a moderate and negative correlation was observed between *p*CA and diferulate DFA 8–5. No significant correlations were observed between grain yield or saccharification efficiency and hydroxycinnamates content. Digestibility of the organic matter was negatively correlated with *p*CA and moderately positive correlated with diferulates (Table 2).

**Table 2:**
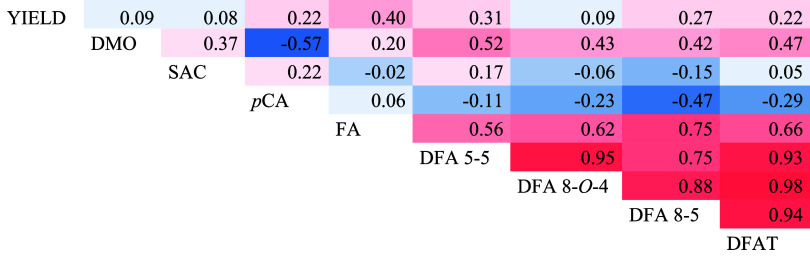
Estimates of the Genotypic Correlation Coefficient for the Hydroxycinnamates Esterified in the Cell Wall and with Grain Yield (YIELD), Digestibility of the Organic Matter (DMO) and Saccharification Efficiency (SAC)^a^

a Color coding: Higher positive values are represented in red, those close to 0 in white, and lower negative values in blue. *p*CA: coumarate; FA; ferulate; DFAT: total diferulates.

### Association Analysis

The Bonferroni correction for declaring a single comparison significant (*p* < 0.05/number of SNPs) was applied in this study with 153,009 markers, meaning that a marker would be significantly associated with a trait when the *p*-value of that association is less than 3.27 × 10^–7^. However, this approach is considered excessively conservative since all markers are not independent. Consequently, the identified associations are highly significant, suggesting that they have a solid foundation and are unlikely to be spurious or false associations. Additionally, to delimit the regions of interest, a confidence interval around each significant SNP was estimated, which in no case exceeded ±1 Mb (Table 3). When the confidence intervals of two SNPs overlap, they are grouped into the same QTL.

**Table 3:** Significant Markers Associated with Cell Wall-Bound Hydroxycinnamates in 238 Inbreds Evaluated in 2018 and 2019

trait	QTL^a^	marker^b^	QTL interval^c^	Chr	pos^d^	*p*-value	MAF^e^	effect^f^
coumarate	qPCA _2_1	S2_148561880	±1 Mbp	2	153144503	4.72 × 10^–08^	0.314	–0.33
	qPCA _6_1	S6_158227285	±500 kbp	6	162392523	2.43 × 10^–09^	0.224	–0.45
ferulate	qFA_3_1	S3_215758011	±1 Mbp	3	219356964	4.58 × 10^–09^	0.214	0.18
	qFA_5_1	S5_92292823	±1 Mbp	5	94649392	1.84 × 10^–07^	0.244	–0.17
DFA 5–5	qDFA55_2_1	S2_203613655	±1 Mbp	2	210185813	2.67 × 10^–10^	0.118	0.018
	qDFA55_5_1	S5_1042978	±1 Mbp	5	1081286	8.12 × 10^–10^	0.402	–0.012
	qDFA55_5_2	S5_4587576	±1 Mbp	5	4693619	9.58 × 10^–11^	0.177	0.018
	qDFA55_5_3	S5_191376676	±1 Mbp	5	196743533	4.28 × 10^–09^	0.252	–0.014
	qDFA55_7_1	S7_141427986	±100 kbp	7	146343121	2.04 × 10^–08^	0.256	0.012
	qDFA55_9_1	S9_90843307	±1 Mbp	9	94210342	1.04 × 10^–09^	0.274	–0.013
	qDFA55_10_1	S10_130304439	±1 Mbp	10	131495139	1.67 × 10^–09^	0.115	–0.019
	qDFA55_10_2	S10_143164369	±100 kbp	10	143881438	1.80 × 10^–07^	0.194	–0.013
DFA 8-*O*-4	qDFA8O4_8_1	S8_114779212	±500 kbp	8	117532075	2.55 × 10^–08^	0.342	–0.018
total diferulates	qDFAT_7_1	S7_173516190	±500 kbp	7	177374565	5.68 × 10^–08^	0.167	0.062
	qDFAT_9_1	S9_129048422	±1 Mbp	9	131293869	4.03 × 10^–08^	0.235	0.055

a qPCA: QTL for coumarate; qFA: QTL for ferulate; qDFA55: QTL for diferulate DFA 5–5; qDFA8O4: QTL for diferulate DFA 8-*O*-4; qDFAT: QTL for total diferulates. The number after the 1st underscore indicates the chromosome, and the number after the 2nd underscore indicates the QTL within the chromosome.

b The number before the underscore indicates the chromosome and the number after the underscore indicates the position of the marker in B73 Reference Genome version 2 (pb).

c Confidence interval based on the distance from which linkage disequilibrium exhibits *r*^2^ values <0.2.

d Physical position of the marker on the chromosome in version 4 of the B73 sequence (Zm-B73-REFERENCE-GRAMENE-4.0).

e MAF: Minor allele frequency.

f Additive effect calculated as half the difference between the mean of the homozygote for the second allele in alphabetical order and the mean of the homozygote for the complementary allele.

**Figure 1:**
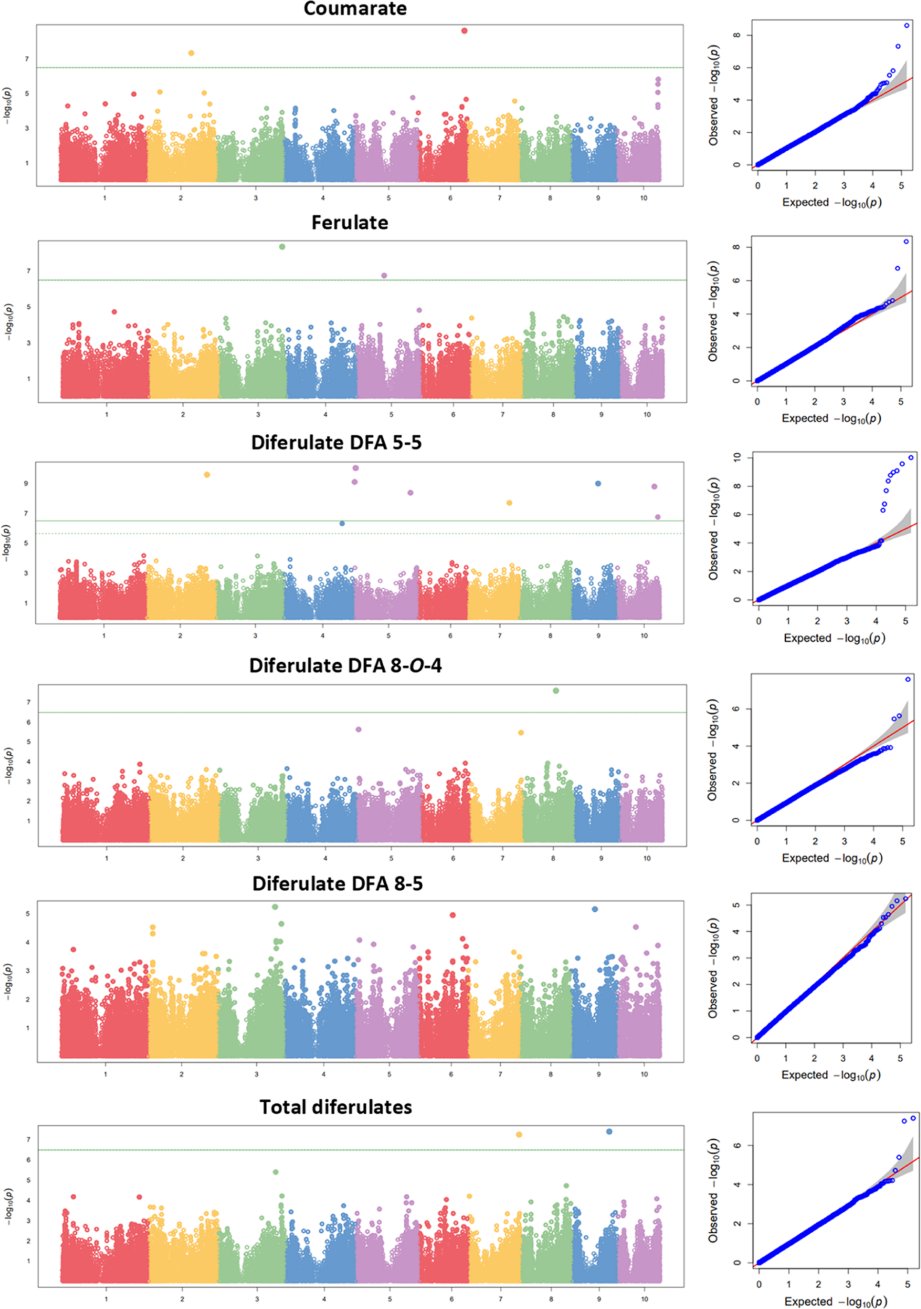
Manhattan and quantile-quantile (QQ) plots for cell wall-bound hydroxycinnamates of 238 lines from the Ames Association Panel evaluated in Pontevedra in 2018 and 2019. Single nucleotide polymorphisms (SNPs) that are above the green horizontal line in the Manhattan plot exceed the Bonferroni threshold set for an experiment-wide error of 0.05. In the QQ plot, shaded lines represent the 95% confidence interval under the null hypothesis of no association between the SNP and the trait.

A total of 15 significant SNPs were identified, none of which corresponded to the same QTL (Figure 1; Table 3). Of the 15 significant markers, 2 are associated with pCA, 2 with FA, 8 with the DFA 5–5, 1 with the DFA 8-*O*-4, and 2 with DFAT. DFA 5–5 is the trait for which most significant markers have been identified. SNP S2_203613655 and SNP S5_1042978 exhibit *p*-values lower than 1.00 × 10^*–*10^, and marker S5_4587576 had the most significant association with a p-value of 9.58 × 10^*–*11^ for its association with DFA 5–5.

The search for candidate genes was performed within the confidence intervals of each significant SNPs and are shown in Supporting Information. Genes with annotated functions possibly related to the hydroxycinnamate content in the cell wall were highlighted. We found 1 candidate gene associated with *p*CA content, 2 with DFA 5–5, and 1 with DFA 8-*O*-4. Within the confidence interval of QTL qPCA_2_1, we discovered the gene *Zm00001eb093590*, which encodes for a 4-coumarate--CoA ligase (EC 6.2.1.12). For DFA 5–5, we identified gene *Zm00001eb106090* in the qDFA55_2_1 interval and *Zm00001eb212660* in the qDFA55_5_1 interval, with the annotated functions of Cytochrome P450 family 718 and GALT4 hydroxyproline O-galactosyltransferase, respectively. Finally, in qDFA8O4_8_1, the gene *Zm00001eb350960* is a UDP-glycosyltransferase 88A1.

### Genomic Prediction

**Figure 2:**
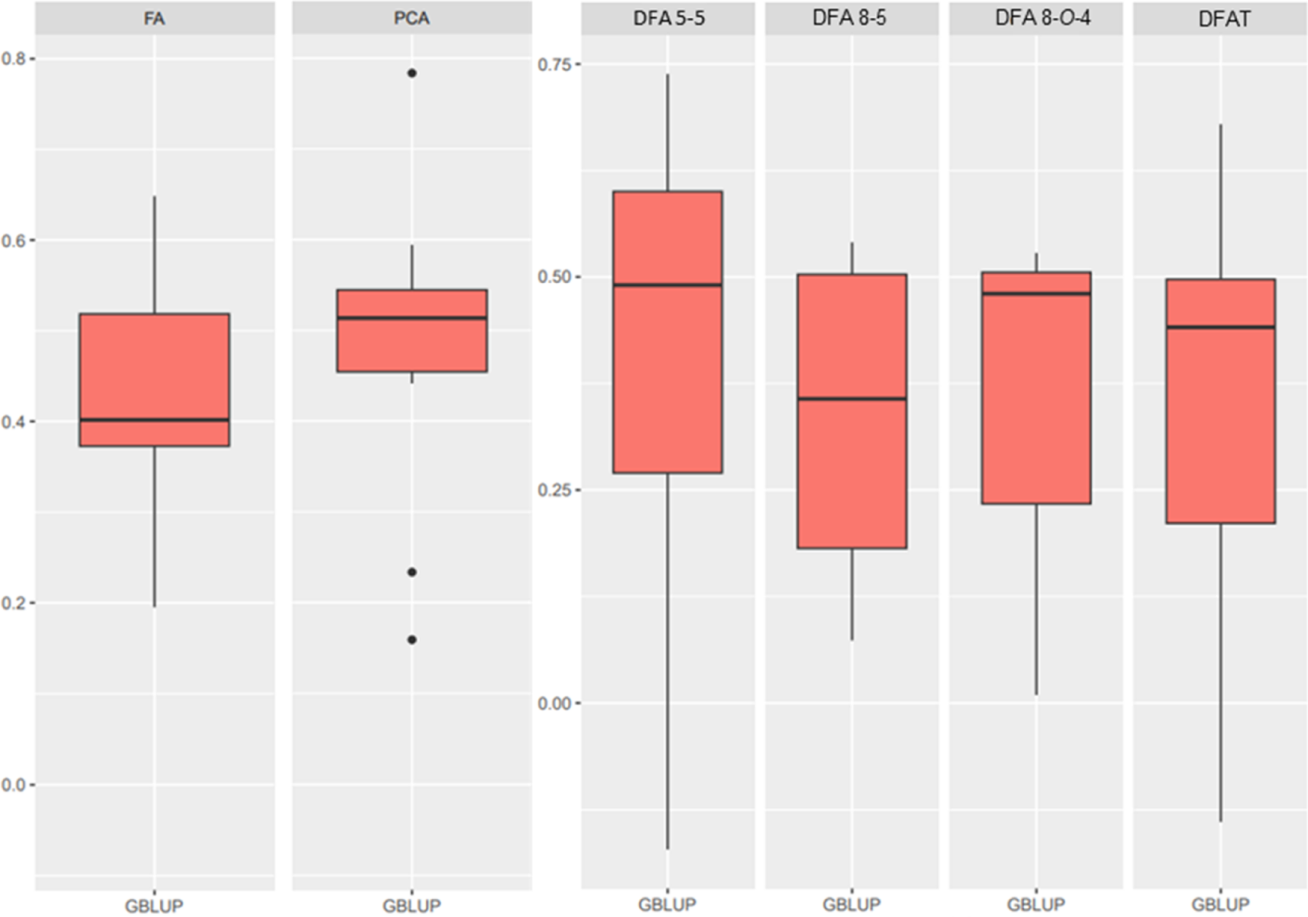
Comparison of predictive capacity and its standard errors averaging 10 cross-validation iterations following the GBLUP model for cell wall-bound hydroxycinnamates. FA: ferulate; PCA: coumarate; DFA 5–5: diferulate DFA 5–5; DFA 8–5: diferulate DFA 8–5; DFA 8-*O*-4: diferulate DFA 8-*O*-4; DFAT: total diferulates.

We predicted the genetic values of each inbred line and evaluated the predictive capacity and accuracy of the prediction for all traits by using the GBLUP model. For cell wall-bound hydroxycinnamates, a higher predictive capacity is observed in the GBLUP model for *p*CA (0.45), followed by ferulate and diferulates, which have values around 0.35. The accuracy across all traits is approximately 0.50, suggesting that the model has a moderately high capacity for making accurate predictions for hydroxycinnamate contents (Figure 2; Table 4).

**Table 4:** Predictive Ability, Accuracy, and Standard Error of Predictive Ability Based on the Variance of the 10 Prediction Folds (SE) of the GBLUP Model for Cell Wall Hydroxycinnamates

trait	predictive ability	accuracy	SE
coumarate	0.45	0.54	0.04
ferulate	0.35	0.44	0.06
DFA 5–5	0.39	0.54	0.09
DFA 8-*O*-4	0.36	0.53	0.07
DFA 8–5	0.34	0.48	0.06
total diferulates	0.36	0.52	0.08

## Discussion

### Genome-Wide Association Study

The genetic diversity within the studied population is underscored by the significant differences observed among the various inbred lines regarding the content of cell wall-bound hydroxycinnamates.^27^ The moderately high heritability estimates are consistent with results obtained in previous research.^2,16,50^ Although the heritability estimates suggest that phenotypic selection could be effective, it is important to note that biochemical analysis of hydroxycinnamates requires more time and resources compared to directly measurable traits in the field.^51^

The strong correlations between cell wall components follow the pattern reported in previous studies.^16,17,50^ This implies that if the aim is to improve FA, individual and total dimers will also be affected and the vice versa. Additionally, the lack of significant correlations with grain yield indicates that enhancing these traits should not negatively impact the plant yield. However, this does not apply to *p*CA, as it shows no correlation with any other trait. The significant genotypic correlations between FA and diferulates support the search for colocalizations between these traits within this study. However, such colocalizations were not found, presumably due to the polygenic nature of these traits, where most of the genetic variation is not associated with individual SNPs.

We also observed no cases of colocalization between the identified QTLs in this study for each hydroxycinnamate with those identified for the same hydroxycinnamates in a different inbred panel of 270 inbreds.^16^ This lack of colocalization among QTLs identified for the same trait may stem from the fact that the previous study quantified cell wall-bound hydroxycinnamates in the pith of specific stem internodes, whereas we used the entire stover (whole plant without ears) for hydroxycinnamate quantification. As genes regulating plant secondary metabolism are typically tissue-specific, the different tissues used in both studies may influence the lack of QTL colocalizations.^15^ In a more recent GWAS study on hydroxycinnamates in a multiparent advanced generation intercross (MAGIC) population, carried out by the same group but using maize stover samples, authors found QTL that colocalize with QTL detected in the current study.^50^ The QTLs qDFA5–5_5_1 and qDFAT_5_1 associated with DFA 5–5 and DFAT in the study by López-Malvar et al.^50^ deserves special attention as it was localized less than 1 kb apart from the QTL qDFA55_5_2 found in the current study. These findings highlight the importance of this genomic region as an especially interesting area for the identification of candidate genes probably involved in diferulate accumulation.

In the confidence interval of qDFA55_5_2, a gene with an annotated function of the Cytochrome P450 (CYP) family 718 has been identified. This family plays a crucial role in several metabolic pathways, although the specific function of the CYP enzyme family 718 has not yet been fully characterized. Although most of the functions of CYP family enzymes are not fully understood, they are known to be involved in a wide range of metabolic activities, including oxidation, reduction, and/or hydrolysis of various molecules.^52,53^ In this particular context, diverse P450s catalyze reactions of the general phenylpropanoid pathways, such as, for example, the related function ferulate-5-hydroxylase (F5H),^54^ but further research is needed to pinpoint its precise function in maize.

In addition, another potential gene related to DFA 5–5 content has been identified in QTL qDFA55_5_2, galactosyltransferase O-hydroxyproline GALT4. This gene is involved in the biosynthesis of arabinogalactan (AGP), a class of plant-specific glycoproteins. Although the GALT4 gene is not directly involved in FA synthesis or dimer formation, its role in AGP biosynthesis suggests an indirect connection to cell wall modification. Since AGPs are key glycoproteins in the structure and function of the cell wall, the activity of the GALT4 gene could influence the composition, which in turn could affect the formation of this diferulate in the cell wall matrix.^9,55^ The UDP-glycosyltransferase 88A1 gene could be a candidate for QTL qDFA8O4_8_1 associated with DFA 8-*O*-4 content. This enzyme, UGT88A1, is a sugar-dependent glycosyltransferase, first identified in *Arabidopsis thaliana*.^56^ Although its functions are not fully understood, previous research indicates that this enzyme may be involved in the conversion of compounds such as quercetin in the phenylpropanoid pathway and may influence cell wall synthesis and properties.^56,57^ In addition, it has been observed that glycosyltransferases can divert the flavonoid metabolic pathway toward the production of other phenolic compounds, which could represent a new approach to manipulate phenolic acid synthesis in plants.^58^

Finally, the confidence interval of QTL qPCA_2_1 includes a 4-coumarate:CoA ligase, one of the key branch point enzymes in the phenylpropanoid pathway. The 4-coumarate:CoA ligase enzyme family (4CL; EC 6.2.1.12) catalyzes the activation of 4-coumarate and related substrates to form the respective CoA esters, thus directing the common building block derived from phenylalanine toward different branches of the phenylpropanoid metabolism. These phenylpropanoid branch pathways generate various classes of natural secondary compounds with essential functions in plant development and environmental interactions, such as lignin for structural support and resistance of the plant.^59^

Pest damage or resistance traits have not been assesed in this panel, although previous studies highlighted evidence on the role of hydroxycinnamates in maize resistance to stem tunneling by borers,^9,60^ and suggest that the role of diferulates in cross-linking hemicellulose chains, binding specifically to arabinoses, could lead to greater pest resistance.^61^ A colocalization was found between QTL qDFA55_5_1 associated with DFA 5–5 and SNPs S5_1442632 and S5_1848216 identified by Jiménez-Galindo et al.^62^ for resistance to stem tunneling by borers. Furthermore, if this concept is extended to other pests, such as *Fusarium* fungal genera, colocalizations were identified between QTL qDFA55_10_1 and qDFA55_7_1 associated with DFA 5–5 in this study and fumonisin content in maize grain in a MAGIC population.^63^ This highlights the possible role of 5–5 diferulates in pest resistance. However, a more rigorous validation of these findings is needed to confirm their significance, so it is suggested that a resistance assessment be carried out in this association panel to see what correlation there is between resistance and diferulates content.

In relation to animal digestibility and saccharification efficiency, significant genotypic correlations have been identified only between digestibility and *p*CA or diferulates. This significant association suggests the possibility of identifying colocalizations using the same material within the same panel; however, no QTLs significantly associated with digestibility of organic matter (DMO) have been detected, neither colocalizations between hydroxycinnamates content with acid and neutral detergent fibers,^37^ traits typically linked to animal forage quality.^64^ The lack of association in genetic studies can be attributed to the inherent complexity of the traits studied, which are polygenic, and where the identified loci explain only a fraction of the observed variability. In addition, genetic correlations may be influenced by genomic regions that were not identified as relevant.

In deep, the genetic correlation found between DMO and *p*CA is negative, while it is positive with diferulates content, indicating that the diferulates content is associated with higher DMO. Previous research has indicated that diferulic esters form a polysaccharide matrix that is resistant to cross-linking of hemicellulose chains, which hinders animal digestibility.^13,17^ However, this observation may be specific to the analyzed tissue, the pith, where such previous studies were conducted, and may not be extrapolated to stover. Alternatively, the incorporation of laccases/peroxidases into the wall matrix, which could catalyze the dimerization of FA, could also interfere with lignin polymer formation, providing another level of control over the lignification process.^65^ This finding suggests that a wall with a higher content of diferulate-mediated cross-links may confer certain pest resistance to the plant, but also a lower lignin content that delays animal digestibility.^66^ Finally, we also have to note that a portion of FA and DFA could become undetectable by the method used in this study because it cross-links to lignin via ether or C–C bonds. The ether fraction could play a proper negative role in digestibility, but harsher alkali solvolytic methods used in some of these analysis could underestimate, to an unknown degree, the extent of FA- and DFA-lignin cross-linking in lignified tissues.^3,13,67^

On the other hand, the negative coefficient between *p*CA and DMO might suggest that the acylation of S-subunits by *p*CA previously mentioned exerts a significant influence on how lignin S-units bind, spatially organize, and interact with polysaccharides. This agrees with an obstruction of the cell wall breakdown, which could be associated with reduced digestibility.^68^

The negative genetic correlation of −0.57 between DMO and *p*CA suggests that reducing the latter would indirectly increase DMO. As a result, the concentration of *p*CA could be used as an indirect indicator to enhance digestibility. Additionally, by decreasing the *p*CA concentration and increasing DMO, the concentration of diferulate 8–5, which has a negative correlation of −0.47 with *p*CA, could potentially increase. Thus, reducing *p*CA could elevate both DMO and diferulates levels. This strategy could be intriguing for improving digestibility. However, phenotypic selection for reducing *p*CA does not seem to be an appealing option since DMO is easier to measure phenotypically than *p*CA, and both have moderate heritability.^37^ On the other hand, the predictive capacity of DMO in genomic prediction is very low.^37^ Therefore, it would be viable to consider indirect genomic selection using pCA as a key trait.

### Genomic Prediction

As previously mentioned, although moderate heritability suggests that phenotypic selection could be effective, this has been characterized by being costly, labor-intensive, and time-consuming. With the decreasing costs of genotyping techniques, marker-assisted selection has emerged as a more economically efficient alternative, whether using a few markers or the whole genome. Nevertheless, the study of QTL involved in the inheritance of each hydroxycinnamate provides information about the complexity of its inheritance, revealing that all are polygenic. Therefore, genomic selection appears to be a promising alternative, considering that the successful integration of genomic selection into the breeding process depends largely on factors such as the cost and time associated with phenotyping as well as the ability to predict the phenotype based on genotypic data.

We observed that the predictive capacity of GBLUP genomic models was moderate for all studied hydroxycinnamates, supporting the effectiveness of using this panel, which covers a wide range of genetic material,^26^ as a generic training set for maize cell wall hydroxycinnamates. The moderate predictive ability for these traits can be attributed to the heritability of the trait, the experimental design, the marker density, the size of the training population, the within-population variability of the trait, and the relationship between the training and test populations.^69^ Previous studies have shown that genomic prediction leads to high correlations between predicted and observed genetic values for a quantitative trait when the predictive capacity of the model is moderate.^22^

Based on the observed results, the genomic selection approach could prove to be a valuable tool for parental selection at the beginning of breeding processes, thereby avoiding the need for extensive phenotyping. However, the use of hydroxycinnamates as an indirect selection method in stover requires further analysis. Although the biochemical and/or structural characteristics influenced by hydroxycinnamates in the maize pith are related to degradability or pest resistance,^9,60^ no such clear effect is observed focusing properly in stover residue. We could advance in this line selecting inbred lines with low *p*CA and high diferulates content to study their effect on the digestibility of stover cell walls, thereby aiding our understanding of their influence on cell wall reinforcement. This highlights again the importance of the tissue under study regarding its final application.

## Data Availability

Data are publicly available at Panzea (http://www.panzea.org). The data sets used and/or analyzed during the current study will be available upon request to Dra. Noemi Gesteiro (ngesteiro@mbg.csic.es).

## Supporting Information Available

qPCA _2_1; qPCA _6_1; qFA_3_1; qFA_5_1 (XLSX)
